# The future of genomic medicine education in Africa

**DOI:** 10.1186/s13073-015-0175-x

**Published:** 2015-05-23

**Authors:** Geoffrey H Siwo, Scott M Williams, Jason H Moore

**Affiliations:** Institute for Biomedical Informatics, Perelman School of Medicine, University of Pennsylvania, Philadelphia, PA 19104 USA; Department of Genetics, Geisel School of Medicine, Dartmouth College, Lebanon, NH 03756 USA; IBM TJ Watson Research Center, Yorktown Heights, NY 10598 USA; IBM Research-Africa, Sandton, 2196 South Africa

## Abstract

There are many challenges and opportunities for Africans in the emerging area of genome medicine. In particular, there is a need for investment in local education using real-world African genetic data sets. Cloud-based computing platforms offer one solution for engaging the next generation of biomedical scientists in tackling disease in Africa, and by extension, the world.

## Challenges for Africa in the genomic medicine era

Africa faces a huge burden of diseases caused by infectious agents, including emerging viral diseases such as the recent Ebola outbreak [1]. In addition, the prevalence of non-communicable diseases in Africa is on the rise, in part due to increasing urbanization. Modern human populations originated in Africa, and as a consequence, Africans have extremely high genetic diversity [2], which may lead to huge variations in disease susceptibility or resistance, drug metabolism and prevalence of adverse side effects [3]. Therefore, genomic information could potentially have a greater impact on the prevention, diagnosis and treatment of diseases in Africa than in many other parts of the world.

Genomic medicine is one of the fastest-growing fields. In the past decade, the amount of genomic information that is available has grown rapidly because of the falling cost and increasing efficiency of DNA sequencing technologies. It took nearly 1 billion US dollars and more than 10 years to complete the sequence of a single human genome in 2002. Today, an individual human genome can be sequenced for less than 5,000 US dollars in one day. In spite of this progress, DNA sequencing is still relatively expensive for large-scale studies and Africa lags behind other continents in carrying out such studies. Both the UK and the US have, in the past 2 years, initiated plans for large-scale genome-sequencing studies that will involve participants numbering in the hundreds of thousands (as in the 100,000 Genomes Project led by Genomics England) to millions (as in the Precision Medicine Initiative recently announced by the US President Barack Obama and led by the National Institutes of Health (NIH)). Although unable to match the scale of these efforts, African scientists are continuously making efforts to perform large-scale genome-sequencing studies that are focused on specific diseases. The H3Africa project, funded by NIH and The Wellcome Trust, is already supporting several studies involving collaborative centers within the continent. Collectively, these studies could generate genome sequence data from 50,000 to 75,000 Africans [4]. For example, the Collaborative African Genomics Network (CAfGen), one of the H3Africa funded bodies, is investigating genomic factors that influence HIV and tuberculosis disease outcomes. Another ongoing large-scale effort in collecting African genetic data is the African Genome Variation Project based at the Sanger Institute. Recently, this project identified new loci associated with malaria susceptibility and hypertension on the basis of the genotyping of 1,481 individuals from 18 enthnolinguistic groups in sub-Saharan Africa and the complete genome sequences of 320 individuals from Ethiopia, Uganda and southern Africa [5]. The African Genome Variation Project has generated the most extensive African genetic diversity resource. Another ongoing large-scale genome-sequencing project in Africa is the MalariaGEN project, which is primarily focused on characterizing genetic variation in the human malaria parasite *Plasmodium falciparum*, genetic diversity in the mosquito vector, and host genetic factors that influence susceptibility and resistance to malaria infection [6].

The future of genomic medicine in Africa will also be determined by the availability of highly skilled individuals in the field. Fortunately, the ongoing genomic projects in Africa are both establishing infrastructure for genomics research and training local researchers, as well as generating genomic datasets. Indeed, many of these projects have made capacity building one of their core missions. In particular, H3Africa has established a bioinformatics network (H3ABioNet), whose core mission is to develop bioinformatics capacity throughout the continent and to provide infrastructure to support genomic analysis and data storage [7]. H3ABioNet members include 32 research institutions located in 15 African countries and two partner institutions in the US [8]. The network is providing training to several students through workshops and internships in areas such as population genetics, genetic epidemiology and data analytics. The Wellcome Trust periodically organizes workshops, such as the Genomic Epidemiology of Malaria course, that provide an opportunity for young researchers in Africa to learn skills in the computational analysis of genomic datasets from leading scientists in the field. H3Africa has developed a data-sharing policy framework that ensures that Africa-based researchers have access to genetic datasets from the project for a period of up to 23 months before the datasets become widely available [9].

## Next steps for genome medicine education: United Genomes Project

The existing genomics projects and capacity-building frameworks are playing a vital role in engaging young scientists across Africa. Nevertheless, scaling genetic data collection across Africa’s diverse ethnicities and providing hands-on training to hundreds or even thousands of students and researchers across the continent remains a challenge. For African scientists to take advantage of the increasing availability of genomic datasets from the continent and for data-sharing policy frameworks that give African researchers first access to the datasets [9], there is a need for affordable computational infrastructure and training opportunities.

The United Genomes Project, first publicly announced at the Technology, Entertainment and Design (TED) Global Conference (Rio de Janeiro, 2014), is developing scalable approaches to meet these needs. It provides an education and open science platform to train students and biomedical scientists in Africa in genomic medicine by engaging them in computational projects, which address defined challenges that are relevant to the continent [10]. The project will help researchers to tackle real-world issues in genomic medicine by: developing scalable approaches to collate genomic data across multiple African ethnicities; building capacity across the continent using cloud computing and interactive programming interfaces; and facilitating scientific discovery through crowdsourcing and open innovation. United Genomes will collate genetic data from multiple ethnic groups from two main sources: first, anonymized data shared by African immigrants to the US who have undergone direct-to-consumer genetic testing provided by 23andMe, and second, collation of all published and publicly available genetic data from African individuals scattered across online repositories. The project will provide access to these datasets (with the level of access to users dependent on the consent given by participants in each study), leverage innovative methods for internet connectivity, and make high-performance cloud-computing resources available to researchers or students so that they can run memory-intensive genomic analyses for research and education without the need to own powerful computers. United Genomes will collaborate with existing educational programs at universities or with programs offered by projects such as H3Africa. For example, researchers at universities or institutes providing teaching programs in Africa will be allowed to create their own courses using data and computational resources available on the project’s website and course materials from H3ABioNet.

The vast majority of educational systems, including the Massive Open Online Courses offered by some universities and other e-learning organizations, all focus on imparting skills to students, which in many cases are expected to be applied by the students in the future. What if education could be realized in parallel with solving a real world problem, using real-world data and expertise? We hope that a cloud-based system for genomic medicine could help train the next generation of biomedical scientists in Africa while enabling them to contribute new knowledge, and importantly, to help solve some of Africa’s medical challenges. If genomic medicine becomes a cornerstone of medicine, the growing gap in knowledge relating to African and European genetics could perpetuate future health disparities. The time for avoiding these disparities is now, when genomic medicine is still in its infancy. The large-scale generation and analysis of African genetic datasets will have a huge impact on medicine in other world populations, just as it has had on expanding our knowledge of the origin and diversity of the human species.
