# Erratum to: The future of genomic medicine education in Africa

**DOI:** 10.1186/s13073-015-0193-8

**Published:** 2015-08-03

**Authors:** Geoffrey H. Siwo, Scott M. Williams, Jason H. Moore

**Affiliations:** Institute for Biomedical Informatics, Perelman School of Medicine, University of Pennsylvania, Philadelphia, PA 19104 USA; Department of Genetics, Geisel School of Medicine, Dartmouth College, Lebanon, NH 03756 USA; IBM TJ Watson Research Center, Yorktown Heights, NY 10598 USA; IBM Research-Africa, Sandton, 2196 South Africa

## Erratum

It has come to our attention that during the production of this article [1], the name of the first author, Geoffrey H Siwo, was incorrectly spelt as Siw. This has been corrected in the online version of the article.
